# A Comparative Assessment of the Pathogenic Potential of Newly Discovered Henipaviruses

**DOI:** 10.3390/pathogens13070587

**Published:** 2024-07-16

**Authors:** Kristina Meier, Judith Olejnik, Adam J. Hume, Elke Mühlberger

**Affiliations:** 1 Department of Virology, Immunology and Microbiology, Chobanian & Avedisian School of Medicine, Boston University, Boston, MA 02118, USA; krmeier@bu.edu (K.M.); jolejnik@bu.edu (J.O.); hume@bu.edu (A.J.H.); 2 National Emerging Infectious Diseases Laboratories, Boston University, Boston, MA 02218, USA

**Keywords:** henipaviruses, Nipah virus, Hendra virus, Cedar virus, Langya virus, Ghana virus, Mojiang virus, emerging viruses

## Abstract

Recent advances in high-throughput sequencing technologies have led to the discovery of a plethora of previously unknown viruses in animal samples. Some of these newly detected viruses are closely related to human pathogens. A prime example are the henipaviruses. Both Nipah (NiV) and Hendra virus (HeV) cause severe disease in humans. Henipaviruses are of zoonotic origin, and animal hosts, including intermediate hosts, play a critical role in viral transmission to humans. The natural reservoir hosts of NiV and HeV seem to be restricted to a few fruit bat species of the *Pteropus* genus in distinct geographic areas. However, the recent discovery of novel henipa- and henipa-like viruses suggests that these viruses are far more widespread than was originally thought. To date, these new viruses have been found in a wide range of animal hosts, including bats, shrews, and rodents in Asia, Africa, Europe, and South America. Since these viruses are closely related to human pathogens, it is important to learn whether they pose a threat to human health. In this article, we summarize what is known about the newly discovered henipaviruses, highlight differences to NiV and HeV, and discuss their pathogenic potential.

## 1. The Classical Henipaviruses

The *Henipavirus* genus belongs to the *Paramyxoviridae* family within the order *Mononegavirales*. A key feature of mononegaviruses is the non-segmented negative-sense RNA genome that, along with the viral nucleocapsid proteins, forms a ribonucleoprotein complex. All mononegaviruses encode an RNA-dependent RNA polymerase, and the virions are enveloped by a host cell-derived membrane. The first henipavirus to be identified and isolated was Hendra virus (HeV; *Henipavirus hendraense*) in 1994, followed by Nipah virus (NiV; *Henipavirus nipahense*) in 1999, both of which became eponymous for this virus genus.

The discovery of HeV in Hendra, a suburb of Brisbane, Australia, was prompted by an outbreak of severe respiratory disease in horses. Two humans, who were in close contact with the sick horses, became ill as well, and one of the patients succumbed to the disease. The causative agent, HeV, was found to be distantly related to morbilliviruses, another genus within the *Paramyxoviridae* family [1,2]. To date, seven cases of human HeV infection have been reported, four of which were fatal [3]. Transmission to humans has occurred through contact with infected horses that were exposed to excretions of infected fruit bats of the *Pteropus* genus, which serve as major natural reservoirs for both HeV and NiV [4,5]. There are no reports of human-to-human transmission or bat-to-human transmission of HeV. A second genotype of HeV (HeV-g2) was recently identified in fruit bats of the *Pteropus* genus and in a horse that showed symptoms of HeV infection but initially tested negative in a PCR targeting HeV-g1 [6,7,8,9].

NiV was identified following an outbreak of severe febrile encephalitis among pig farmers and febrile respiratory disease in pigs in Malaysia and Singapore in 1998–1999. This outbreak, caused by the NiV strain Malaysia (NiV-M), resulted in at least 265 human cases of encephalitis with 105 deaths, and spread to Singapore via infected pigs imported from Malaysia [10,11,12]. Pigs seemed to play an important role in transmitting the virus to humans in this outbreak. Since 2001, a second NiV strain, NiV Bangladesh (NiV-B), has caused nearly annual outbreaks in Bangladesh and India with case fatality rates of around 70%. In contrast to the HeV and NiV-M outbreaks, no intermediate host was identified bridging NiV-B infections in bats and humans, although a potential role of domestic ruminants was hypothesized in a few cases [13]. An additional NiV outbreak was reported in the Philippines in 2014, which was likely caused by NiV-M or a very closely related strain [14]. The potential routes of NiV zoonotic transmission include via contaminated food, such as raw date palm sap, with the virus possibly transmitted directly from bats to humans, as likely seen in NiV-B outbreaks, or it may be transmitted from bats to livestock via food including fruits contaminated with bat saliva or urine. From livestock, the virus can then be transmitted to humans, as reported for the initial NiV-M outbreak. Human-to-human transmission of NiV-B has been reported and occurs through close contact with an infected patient or their body fluids, including respiratory droplets [15,16,17]. In addition to bats, humans, and pigs, naturally occurring NiV infections have been detected in dogs, cattle, and goats [18,19].

## 2. Nipah and Hendra Virus Disease and Pathogenesis

In humans, HeV and NiV infections cause a severe disease that is characterized by extensive vasculitis in different organs, including the lungs and brain. The low case numbers of HeV disease make it difficult to establish a general characterization of typical symptoms. HeV disease in humans starts with acute respiratory symptoms and may progress to encephalitis in severe cases. No neurological symptoms were involved in the first fatal human case. The patient died of a severe respiratory disease with renal failure [20]. The other three reported fatal cases succumbed to encephalitis, which in one case was a relapse 13 months after recovery from the initial infection [21,22,23]. Of the surviving three patients, two developed a solely respiratory disease while the third patient developed respiratory symptoms and encephalitis and recovered after a prolonged illness [20,22,24].

NiV infection is mainly characterized by severe febrile encephalitis that may involve the brainstem. Respiratory symptoms may occur and appear to be more common and severe in patients infected with NiV-B (70% of patients) than in patients infected with NiV-M (25% of patients) [25]. Gastrointestinal manifestations such as vomiting were also more prevalent in patients infected with NiV-B (58%) compared to patients infected with NiV-M (27%) [25].

Other symptoms include headaches, drowsiness, dizziness, myalgia, seizures, and reduced consciousness [25,26]. It is noteworthy that late-onset neurological symptoms may also manifest years after a non-encephalitic NiV infection. Relapsing encephalitis of recovered patients has been reported a few months to several years after the initial infection [27,28,29,30]. Our knowledge about the causes of persistent NiV infection remains limited, but it should be noted that virus persistence and resulting human disease after initial acute infection has also been reported for other members of the *Paramyxoviridae* family, including the measles virus [31]. Interestingly, abnormal brain scans have also been reported in a small number of seropositive but asymptomatic patients [32].

Various animal models have been established to study HeV and NiV pathogenesis and transmission, including African green monkeys, horses, pigs, bats, cats, dogs, ferrets, guinea pigs, hamsters, and mice, highlighting the exceptionally broad species tropism of these viruses. There are comprehensive reviews that describe and discuss the different aspects of the established animal models [33,34,35,36]. Notably, when bats of the *Pteropus* genus were experimentally infected with NiV or HeV, they showed no signs of disease. The mechanisms underlying the control of henipavirus infection in fruit bats are not well understood. It has been hypothesized that the immune system of bats has evolved to control and tolerate viral infections without showing the typical adverse effects of antiviral immune responses, such as an exacerbated inflammatory response. However, a more thorough understanding of the immune system across bat species is required to be able to dissect viral control mechanisms in reservoir hosts [34,37,38,39,40,41].

## 3. Discovery of Novel Henipaviruses

**Figure 1:**
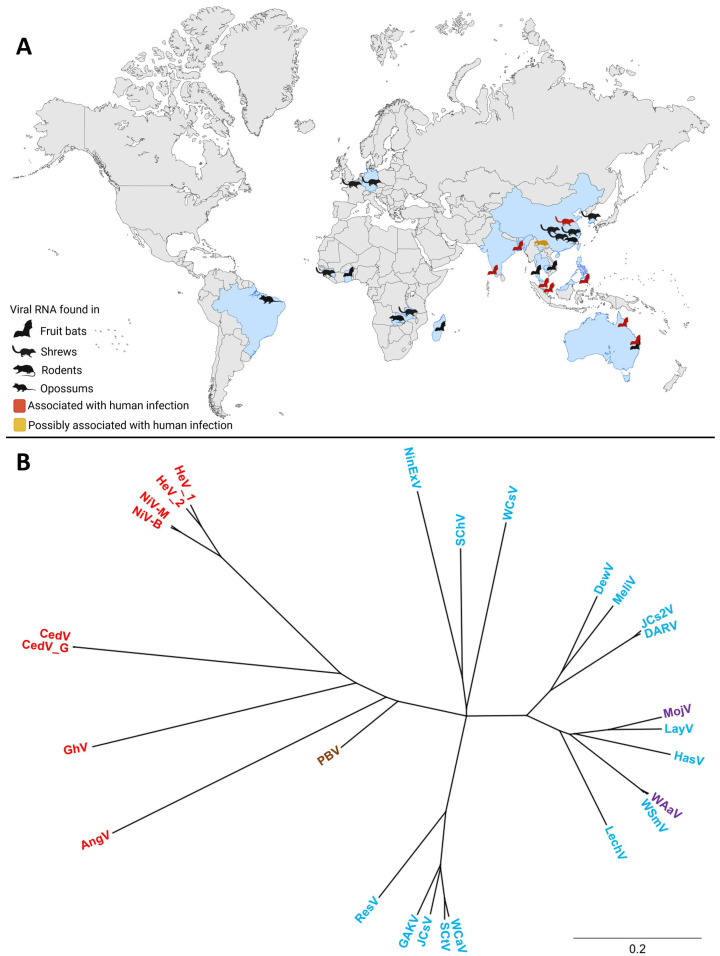
The global distribution and phylogenetic tree of henipa- and henipa-like viruses. (**A**) Countries in which viral genomes or genome fragments with homology to henipaviruses have been reported are indicated in blue. Black animal icons indicate viral RNA-positive samples. Red animal icons indicate outbreaks of human disease. The yellow animal icon represents the discovery of MojV, for which the association with human disease remains unclear. Created with BioRender.com. (**B**) A phylogenetic tree of henipa- and henipa-like viruses using the protein coding sequence of all viral genes. Generated with Geneious Prime. Sequences of the V and W proteins shared in common with the P gene (i.e., N-terminal to the mRNA editing site) were removed to prevent over-representation of this sequence, whereas the sequence for the X protein for Ninorex virus was included due to the flanking transcription start and end signals. The S protein sequences were excluded from this analysis since they are currently only hypothetical and do not contain flanking transcriptional regulatory sequences. Viruses found in bats are in red, viruses found in an opossum in brown, viruses found in shrews in blue, and viruses found in rodents in purple. The scale bar represents nucleotide substitutions per site. Nipah virus Bangladesh strain (NiV-B, AY988601.1), Nipah virus Malaysia strain (NiV-M, NC_002728.1) Hendra virus genotype 1 (HeV-g1, AF017149.3), Hendra virus genotype 2 (HeV-g2, MZ318101.1), Cedar virus (CedV, NC_025351.1), Cedar virus Geelong (CedV-G, KP271122.1), Ghana virus (GhV, NC_025256.1), Angavokely virus (AngV, ON613535.1), Peixe-Boi virus (PBV, MZ615319), Resua virus (ResV, OR713876.1), Gamak virus (GAKV, MZ574407.1), Jingmen *Crocidura shantungensis* virus (JCsV, OM030314.1), Shiyan *Crocidura tanakae* virus (SCtV, OQ970176.1), Wufeng *Crocidura attenuata* virus (WCaV, OM030317.1), Lechodon virus (LechV, OR713879.1), Wenzhou *Suncus murinus* virus (WSmV, OQ715593.1), Wenzhou *Apodemus agrarius* virus (WAaV, MZ328275.1), Hasua virus (HasV, OR713881.1), Langya virus (LayV, OM101125.1), Mòjiāng virus (MojV, NC_025352.1), Daeryong virus (DARV, MZ574409.1), Jingmen *Crocidura shantungensis 2* virus (JCs2V, OM030315.1), Melian virus (MeliV, OK623353.1), Denwin virus (DewV, OK623354.1), Wufeng *Chodsigoa smithii* virus (WCsV, OM030316.1), Sichuan *Chodsigoa hypsibia* virus (SChV, OQ236120.1), and Ninorex virus (NinExV, OQ438286.1) are included.

Since the initial discoveries of HeV and NiV, the henipavirus genus has expanded to include several additional members. In addition, numerous henipa-like viruses have been discovered in the last couple of years. The geographic distribution and diverse animal hosts of the novel and classical henipa- and henipa-like viruses are shown in Figure 1A, and a phylogenetic tree of these viruses is shown in Figure 1B. To date, five viruses are officially recognized as members of the henipavirus genus by the International Committee on Taxonomy of Viruses (ICTV): HeV, NiV, Cedar virus (CedV; *Henipavirus cedarense*), Ghana virus (GhV; *Henipavirus ghanaense*), and Mòjiāng virus (MojV; *Henipavirus mojiangense*) [42]. However, widespread serologic screenings, as well as metagenomics, have led to the discovery of new potential members of this genus (Figure 1A, Table S1).

CedV was isolated after the inoculation of primary bat cell lines with pooled urine samples collected from *Pteropus alecto* and *Pteropus poliocephalus* fruit bats in Cedar Grove in Queensland, Australia, in 2009. This study was part of a surveillance effort on the genetic diversity and dynamics of HeV in Australian fruit bats, but deep sequencing of the genome of this newly isolated virus revealed that it was a novel paramyxovirus, which was most closely related to the two henipaviruses known at that time [43].

GhV was identified in African straw-colored fruit bats (*Eidolon helvum*) in a global, large-scale study aimed to screen fecal, blood, and organ samples from different bat and rodent species for paramyxovirus RNA [44].

The discovery of MojV was prompted by an outbreak of fatal pneumonia among three miners in the Yunnan province, China, in 2012. In an attempt to identify the possible agent causing the disease, Illumina sequencing was carried out on anal swab samples collected from bats, rats, and shrews within the mine, and MojV RNA was detected in samples collected from buff-breasted rats (*Rattus flavipectus*) [45]. To our knowledge, this was the first time that henipaviruses were detected in potential non-bat reservoir hosts. However, it remains elusive if MojV was the causative agent of the disease.

In the following section, we will describe recently discovered, still unclassified viruses that are closely related to the classified henipaviruses. These newly discovered viruses will be referred to as henipa-like viruses. This includes Angavokely virus (AngV), which was discovered through high-throughput sequencing of urine samples from Madagascan fruit bats (*Eidolon dupreanum*) in Madagascar in 2019, after serological evidence had suggested that three different bat species endemic to Madagascar were exposed to henipa-related viruses [46,47].

Langya henipavirus (LayV), which is closely related to MojV, is of particular interest as it was first identified and isolated from a throat swab of a patient presenting with fever in eastern China in 2018. A surveillance study, which monitored febrile patients at three hospitals in the Shandong and Henan provinces in China between 2018 and 2021, identified a total of 35 patients infected with LayV and reported no fatalities. In a subsequent search for the potential animal reservoir, LayV RNA was detected in 27% of the Asian lesser white-toothed shrews (*Crocidura shantungensis*) and Ussuri white-toothed shrews (*C. lasiura*) sampled, identifying these animals as possible hosts for LayV. In addition, antibodies against LayV proteins were detected in goats and dogs. It is not known if the virus was directly transmitted from shrews to humans or via an intermediate host [48,49].

Other newly identified henipa-like viruses include the Gamak virus (GAKV) and Daeryong virus (DARV), identified in Republic of Korea by RT-PCR screening of white-toothed shrews (*C. shantungensis* and *C. lasiura*) for paramyxovirus infection [50], Shiyan Crocidura tanakae henipavirus (SCtV) discovered in Taiwanese gray shrews in China [51], Melian virus (MeliV) and Denwin virus (DewV), which were discovered in large-headed forest shrews (*C. grandiceps*) in Guinea and greater white-toothed shrews (*C. russula*) in Belgium, respectively [52], as well as Ninorex virus (NinExV), which was recently identified in Eurasian pygmy shrews (*Sorex minutus*) in Belgium [53]. In addition to these published results, the nearly complete genomic sequences of several additional henipa-like viruses discovered in several species of shrews in China and Germany and a rodent in China have been deposited into GenBank but have not been described in published manuscripts. These new discoveries highlight the importance of the shrew as an animal reservoir for henipa-like viruses, as recently reviewed in [54]. Of these viruses, only HeV, NiV, CedV, GAKV, and LayV have been isolated.

Fragments of viral genomes resembling henipavirus RNA were also reported in one rodent and eleven shrews in Zambia during a study screening wild rodents and shrews for paramyxoviruses [55]. Additionally, a partial henipavirus-like genome sequence, designated as the Peixe-Boi virus (PBV), was detected in a Brazilian opossum (*Marmosa demerarae*) [56]. The recent discovery of novel henipa-like viruses and the diversity among different species emphasize the need for the reclassification of the genus. Based on phylogenetic analyses, a division into bat-borne viruses and shrew- or rodent-borne viruses has been proposed [53,57].

## 4. Henipavirus Genome Organization

### 4.1. Henipavirus Genome Organization Is Conserved

**Figure 2:**
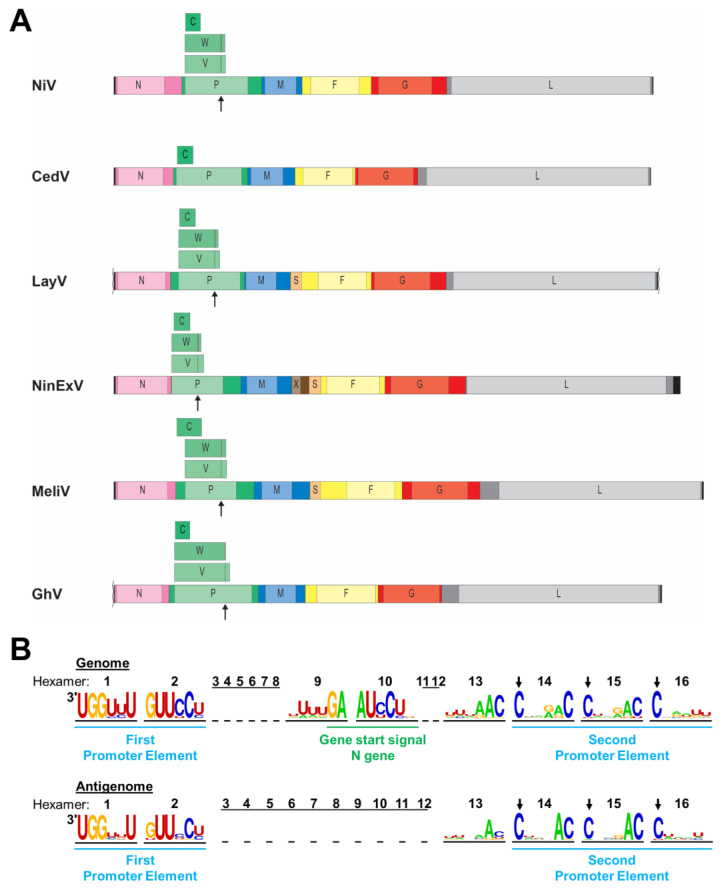
Henipavirus genome organization. (**A**) Schematic diagrams of select henipavirus and henipa-like virus genomes are depicted to scale. Leader and trailer sequences are indicated by black bars, with missing sequence information indicated by squiggly lines (not to scale). Genes are depicted as colored bars with lighter portions indicating open reading frames (ORFs). Additional ORFs encoded by the P gene are depicted above the P gene. Sites of co-transcriptional mRNA editing in the P gene are indicated by arrows underneath the P gene and by lines within the V and W ORFs. N, nucleocapsid protein; P, phosphoprotein; C, V, W, accessory proteins encoded by the P gene; M, matrix protein; X, putative protein of unknown function; S, putative small transmembrane protein encoded within the 5′ UTR of the F gene; F, fusion glycoprotein; G, attachment glycoprotein; and L, RNA-dependent RNA polymerase. (**B**) Nucleotide frequency plot of the bipartite promoter regions located at the viral genome and antigenome ends based on a sequence comparison of 24 henipaviruses and henipa-like viruses. The viral genome and antigenome sequences are depicted in hexamers, and the number of each hexamer is shown above the sequences. The leader region within the genome containing the first promoter element spans nucleotides 1 to 52. The conserved gene start signal of the N gene spans nucleotides 53 to 60. The second promoter element spans hexamers 14 to 16 with the first C residue of each hexamer being conserved (indicated by arrows).

The genome organization is highly conserved among the currently classified bat-borne henipaviruses and resembles that of other paramyxoviruses. Six genes are arranged in a linear order on the non-segmented negative-sense RNA genome, encoding six structural proteins: nucleocapsid protein (N), phosphoprotein (P), matrix protein (M), fusion glycoprotein (F), attachment glycoprotein (G), and RNA-dependent RNA polymerase (L) (Figure 2A). The first gene is preceded by a leader region at the 3′ end, and the last gene is followed by a trailer region at the 5′ end of the genome. The leader comprises the replication promoter required for antigenome synthesis, as well as the transcription promoter for the generation of mRNAs. The complementary trailer (c-trailer) region at the 3′ end of the antigenome contains the replication promoter for viral genome production. In contrast to the leader, the c-trailer does not contain a transcription promoter. The beginning and end of each viral gene is determined by highly conserved gene start and end signals that serve as recognition sites for the viral polymerase to begin and terminate transcription. Each open reading frame (ORF) is flanked by unusually long untranslated regions (UTRs) (Figure 2A). For this reason, the henipavirus genomes are relatively large compared to other paramyxoviruses [58].

Like all paramyxoviruses, henipaviruses follow the “rule of six”, requiring the number of nucleotides in their genome to be a multiple of six for efficient replication [59,60]. The genome is encapsidated by the N protein to form a helical nucleocapsid with each of the N protomers interacting with six nucleotides [61]. The rule of six is also reflected in the structure of the replication promoter (Figure 2B). Henipaviruses have a bipartite replication promoter at the 3′ end of their genomes and antigenomes, respectively. The first promoter element spans the first 12 nucleotides of the 3′ end of the genome or antigenome (hexamers 1 and 2), and the second promoter element comprises hexamers 14 to 16 located in the UTRs of the N or L gene, respectively [58,62] (Figure 2B). For all viruses belonging to the *Orthoparamyxovirinae* subfamily, the first C residue of each hexamer of the second promoter element is conserved [59,63]. However, nucleotide frequency plots of 24 distinct henipa-and henipa-like viruses indicate that additional nucleotides within hexamers 13 to 15 are highly conserved, including a strong preference for a cytosine at position six within these hexamers, and might be part of the replication promoters (Figure 2B). Unlike the leader, the length of the trailer is much more variable, with the L gene stop signal ending as close as 18 nucleotides from the genome end (for CedV, AngV, and WAaV) and as far as 242 nucleotides from the genome end for NinExV.

In addition to the P protein, the henipavirus P gene encodes three accessory proteins, either via an alternative start codon, in the case of the C protein, or via co-transcriptional RNA editing, resulting in the insertion of one or more untemplated G residues in the nascent mRNA strand. This leads to a frame shift, resulting in the synthesis of the V (+G) or W (+GG) protein [64,65,66] (Figure 2A). The V and W proteins share the N-terminal portion with the P protein but have distinct C-terminal regions. These additional proteins are encoded by most identified henipaviruses and have been implicated in immune evasion (see Section 6).

### 4.2. Unique Genome Features of the Newly Discovered Henipa- and Henipa-like Viruses

Unlike all other known henipa-like viruses and other paramyxoviruses, CedV lacks the highly conserved RNA editing site within the P ORF, which is required for the expression of the V and W proteins (Figure 2A). The expression of a C protein via an alternative start codon is conserved, but the position of the C start codon differs among different henipaviruses. While the C start codon of NiV, HeV, CedV, and GhV is located within the borders of the P ORF, it precedes the P ORF in MojV, DenV, MeliV, and GAKV [67] (Figure 2A).

The genome of one of the most recently detected henipa-like viruses, NinExV, appears to harbor an additional gene, encoding a putative protein X, between the M and the F genes. A blastp search did not reveal any similarity with other known sequences, but gene start and end signals were identified for the X gene, indicating that the gene product is likely being expressed [53]. While the X gene has not been previously observed in other henipa-like viral genomes, an additional ORF for a putative small protein (S) upstream of the F gene was first identified in MeliV and DewV and is present in all currently known shrew- and rodent-borne henipa- and henipa-like viruses [52,53,67] (Figure 2A). This ORF might encode a small transmembrane protein of unknown function with a furin cleavage site in the case of LayV [52,67]. Unlike the X ORF, which is flanked by its own gene start and end signals, no gene end and start signals were identified between the S and F ORFs. Therefore, it is not known by which mechanism this putative protein could be expressed [52,67].

## 5. The Role of Henipavirus Receptor Usage in Viral Tropism

### 5.1. Organ and Cell Tropism of NiV and HeV Infections in Humans

There are excellent reviews on NiV and HeV organ and cell tropism in patients, animal models, and cell culture systems [33,35,57,67,68,69,70]. For the purpose of this article, we focus on the infection of humans.

Studies on autopsy specimens from deceased NiV-M-infected patients showed that endothelial cells of the microvasculature in the central nervous system (CNS), lung, heart, and kidney are the main target cells of NiV infections. Vascular infection was not observed in the spleen or liver [26,68,71]. Occasional giant multinuclear, NiV-antigen positive cells within the microvasculature point to syncytia formation of infected endothelial cells [26]. A comparative study on human induced pluripotent stem cell (iPSC)-derived artery and vein endothelial cells showed that artery cells are preferentially infected with both NiV and HeV, due to a higher receptor density compared to vein cells [72]. Autopsy studies of NiV-infected patients who succumbed to acute NiV encephalitis revealed systemic infection of the CNS, as well as non-CNS vascular and extravascular tissue. Viral antigen was observed in the cerebral cortex, brain stem, and cerebellum. There was evidence of direct infection of neuronal, vascular, and parenchymal cells within the CNS. Glial cells (astrocytes and oligodendrocytes) were rarely infected [12,26,73]. The smooth muscle cells that line the tunica media of small blood vessels are also target cells of NiV infection [26,68,73,74].

NiV infections can cause severe respiratory distress in patients. While the microvasculature is also a main target of NiV infection in the lung, it is not clear to what extent epithelial cells of the upper and lower respiratory tract are affected because most of the autopsy samples are from patients who succumbed to acute NiV encephalitis. However, cell culture experiments showed that human primary tracheal/bronchial, small airway, and olfactory epithelial cells are permissive to NiV infection [75,76]. During a NiV disease outbreak in the state of Kerala, India, in 2018, 11 out of 12 patients required ventilatory support, and bilateral infiltrates were visible in the chest X-rays of 9 patients [77]. Notably, except from the index case, all patients from this outbreak contracted NiV through nosocomial transmission. A case study of one of the deceased patients, who had presented with symptoms suggestive of encephalitis, pneumonia, and myocarditis, reported diffuse alveolar damage, the presence of large aggregates in pneumocytes reminiscent of viral inclusion bodies, and the presence of syncytial giant cells in the lung, whereas neuronal, vascular, or myocardial changes were not observed [78]. Natural NiV-M infection of pigs also showed extensive involvement of the respiratory tract, including infection of the upper and lower respiratory epithelium, whereas CNS damage was less pronounced compared to NiV-M-infected patients [28,71].

The cell and organ tropism of HeV in human patients who presented with acute encephalitis is similar to that reported for NiV-M infections [23,28]. HeV infection was also observed in the lung and kidney, specifically in alveolar type 2 epithelial cells, intra-alveolar macrophages, and occasionally, in kidney glomeruli and tubules [23].

Apart from low replication in dendritic cells and the monocytic cell line THP-1, leukocytes do not seem to be susceptible to henipavirus infection, but the virus can attach to non-permissive cells and thus be transported to new sites [79,80,81]. One study reported that dendritic cells and THP-1 cells infected with NiV showed increased transendothelial migration activity compared to mock-infected cells in a model mimicking the blood–brain barrier with human brain microvascular endothelial cells, indicating a possible entry route into the CNS [81].

### 5.2. NiV and HeV Receptor Usage

**Figure 3:**
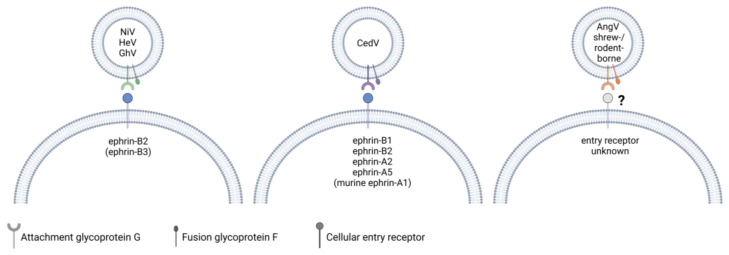
A comparison of the known entry receptors for henipaviruses. NiV and HeV G use ephrin-B2 and, to a lesser extent in the case of HeV, ephrin-B3 as entry receptors. GhV G binds to ephrin-B2 but not -B3. CedV G binds to human ephrin-B1, -B2, -A2, -A5 and murine ephrin-A1. The entry receptors of bat-borne AngV as well as henipa-like viruses within the shrew- and rodent-borne clade are currently unknown. While G recognizes and binds to the cellular entry receptor, both G and F glycoproteins are required for viral and host cell membrane fusion. Created with BioRender.com.

The entry receptors for HeV and NiV are critical determinants of both their host and cellular tropism. The HeV and NiV G protein binds to the cell surface glycoproteins ephrin-B2 and ephrin-B3 (Figure 3), which are expressed in various tissues and organs, including arterial endothelial cells, upper respiratory tract epithelial cells, neurons, and others, contributing to the systemic nature of the disease [82,83,84,85]. The highly conserved sequences of ephrin-B2 and ephrin-B3 among susceptible hosts explain the broad host range of henipaviruses. While both viruses efficiently use ephrin-B2 as entry receptors, NiV G was shown to bind ephrin-B3 with a higher affinity than HeV G. Since ephrin-B3 is widely expressed in the CNS, including the brain stem, which lacks ephrin-B2, this may contribute to brain stem disfunction reported in fatal encephalitis cases caused by NiV [85]. Both the G and F proteins are required for the fusion of the virion with the host cell membrane with F mediating the fusion process. The interaction of virus-infected cells expressing G and F on the plasma membrane with receptor-expressing cells leads to the formation of large multinucleated syncytia, which might contribute to thrombosis, vasculitis, and necrosis [11,26,86].

### 5.3. Receptor Usage of Newly Discovered Henipa- and Henipa-like Viruses

Similar to HeV and NiV, most bat-borne henipaviruses use ephrin proteins for cell attachment. CedV G is unique in that it has an exceptionally broad ephrin tropism. It binds to ephrin-B1, -A2, and -A5 as well as mouse but not human ephrin-A1 (Figure 3). Of the known ephrin receptors, both CedV and GhV G are capable of binding to ephrin-B2 but not ephrin-B3 [87,88,89]. While G recognizes and binds to the entry receptor, F also appears to play a role in determining host tropism, as syncytia formation of GhV G and F expressing cells was found to be restricted to bat cell lines derived from *Hypsignathus monstrosus* and *Eidolon helvum*, although these syncytia contained less nuclei than syncytia induced by co-expression of NiV G and F [90,91,92]. However, syncytia formation was observed in a range of mammalian cell lines when GhV G was co-expressed with NiV F, but not when GhV F was co-expressed with NiV G [91,92]. F needs to be cleaved from a precursor to form a biologically active form, and a study by Weis et al. found that the portion of biologically active surface-expressed GhV F was smaller than it was the case for NiV F in canine cells, which may contribute to the reduced fusogenicity in these cells [92]. Interestingly, the truncation of the GhV G cytoplasmic domain, which is longer compared to that of NiV G, enhanced the fusogenicity of GhV F in *E. helvum*-derived cells and restored GhV F fusogenicity in non-chiropteran cell lines derived from Syrian golden hamsters and African green monkeys. [93]. Notably, although AngV is a bat-borne virus, its G protein lacks conserved ephrin-binding residues, and the entry receptor is currently unknown [46].

To date, the entry receptors of shrew- and rodent-borne henipa-like viruses have not been identified. Structural studies on the highly similar MojV and LayV G proteins showed that the proteins have the characteristic six-bladed β-propeller fold conserved among paramyxovirus G proteins, but the receptor-binding sites differ from that of other henipavirus G proteins and are incompatible with ephrin-B1, -B2, and -B3 binding, which was experimentally confirmed [94,95,96,97,98]. It was also shown that MojV G does not bind to the known cell receptors of other paramyxoviruses, such as CD150 or sialic acid [94]. When compared to HeV and NiV G and F proteins, the overall sequence conservation is higher for MojV and LayV F than it is for G. A structural analysis of MojV and LayV F proteins revealed that the structure of these proteins is overall very similar to that of NiV and HeV F proteins, but the sequence homology is lower for surface-exposed residues [99,100]. In addition, MojV and LayV F proteins differ in their glycosylation pattern from NiV F [99]. Generally, MojV and LayV G and F proteins appear to be less glycosylated than other characterized paramyxovirus glycoproteins [94,99]. These differences might have an important impact on antigenicity, as known neutralizing antibodies binding to NiV and HeV F did not recognize MojV F and LayV F. Similarly, polyclonal NiV F-specific mouse antiserum did not cross-react with either F protein, whereas one of two tested antibodies raised against MojV F reacted with LayV F [96,99]. This indicates that future vaccines or therapeutic interventions targeting the classical bat-borne henipavirus glycoproteins may not be effective against shrew- and rodent-borne henipa- and henipa-like viruses. Although the entry receptors of many novel henipa-like viruses, including LayV, differ from those of the classical henipaviruses, the isolation of LayV from patients suffering from a febrile disease shows that LayV is able to infect humans and cause disease [46].

## 6. The Role of Innate Immune Responses in Henipavirus Pathogenesis

### 6.1. NiV and HeV Suppress Type I Interferon Responses

The activation of the interferon (IFN) response via treatment with dsRNA (poly(I):poly(C12U)) prevented fatal infection in five out of six NiV-infected hamsters, emphasizing the crucial role of the type I IFN system in the host defense against henipavirus infection [101]. Like many other non-segmented negative-sense RNA viruses, the NiV genomic RNA contains 5′ triphosphates that have the potential to activate the type I IFN response through RIG-I signaling [102]. However, both NiV and HeV express multiple proteins that interfere with the induction and signaling of type I IFN (Table S2, reviewed in [34,103,104]). As mentioned above, the NiV and HeV P gene encodes four proteins. This includes P, which is generated by translating the non-edited, full-length ORF of the P transcript. The V and W proteins are generated through mRNA editing and share the N-terminal domain with P. Finally, the C protein is produced by using an alternative start codon within the P mRNA [64,65,66]. All P gene products have been implicated in modulating host innate immune responses, including IFN production and signaling [25,103,104,105,106,107,108]. The NiV and HeV V proteins interact with multiple RNA sensors (RIG-I, MDA5, and LGP2), thereby blocking type I IFN induction in human, murine, bovine, and avian cells [109,110,111,112,113]. NiV and HeV P, V, and W proteins interact with STAT1, STAT2, and STAT4 to inhibit downstream signaling [103,114,115,116,117,118]. NiV V has also been found to interact with STAT5 [119]. NiV C also functions as an IFN antagonist [119].

In addition to the P gene products, NiV and HeV N proteins were shown to suppress both type I and type II IFN responses and block STAT1/2 nuclear translocation [120]. STAT1/2 proteins are also sequestered in viral inclusion bodies during NiV infection, thereby preventing antiviral signaling [121]. Immune modulatory functions were also discovered for the NiV and HeV M proteins, which are able to inhibit both IFN induction and IFN signaling pathways by promoting the degradation of TRIM6 and the subsequent inhibition of Iκκε activation [122].

Although henipaviruses express several proteins counteracting the IFN response, the upregulation of type I and II IFN, IFN-stimulated genes (ISGs), and proinflammatory chemokines and cytokines was observed in infected cell cultures and animal models [39,123,124,125,126,127]. In a recent study, NiV infection led to the nuclear localization of IRF3 and NFκB in infected A549 cells, indicative of the induction of an IFN response. While autocrine type I and III IFN signaling was inhibited in the infected cells, paracrine IFN signaling in neighboring non-infected bystander cells led to the induction of an antiviral response, as reflected by ISG expression [121]. These data suggest that infected and non-infected bystander cells contribute differently to the observed IFN and inflammatory response during HeV and NiV infections.

### 6.2. Correlation between Henipavirus Pathogenicity and Efficient Inhibition of IFN Responses

In contrast to NiV and HeV, CedV is nonpathogenic in guinea pigs, ferrets, hamsters, and mice [43,128,129]. In addition, there are no reported human cases of CedV infection, suggesting that CedV is apathogenic across many species. This attenuated phenotype has been attributed to a reduced ability of CedV to block IFN responses. The CedV P gene lacks the highly conserved RNA editing site found in the P genes of all other known henipa- and henipa-like viruses and does not produce the V and W proteins [43] (Figure 2A). In the hamster model, CedV infection led to the induction of IFN signaling and CXCL10 expression. In contrast, these pathways were suppressed in NiV-infected hamsters [128]. In line with these results, CedV P lacks the ability to inhibit STAT signaling and ISG expression, and STAT1 translocation is inhibited less efficiently in CedV-infected human cells compared to HeV-infected cells [130]. This suggests that modulating the host immune response plays a key role in henipavirus pathogenicity. This is supported by work with recombinant NiV (rNiV) mutants, which did not express V, W, or C. While rNiV mutants lacking V, W, or C expression were still able to block ISG induction in cell culture, differences in pathogenicity were observed in the hamster model. rNiV lacking W was as lethal as wt virus infection, whereas animals infected with rNiV lacking V or C expression showed no clinical symptoms of disease [106]. rNiV lacking C expression showed no markers on inflammation in infected hamster brains, yet increased inflammation was detected in the lungs. However, only wt NiV led to observable necrosis in the lung [131]. In the ferret model, infection with rNiV lacking either C or W remained lethal despite less respiratory distress, whereas a lack of C and W combined led to reduced fatality rates. Ferrets infected with rNiV lacking V expression all survived [125,132]. Interestingly, surviving ferrets infected with rNiV lacking C and W showed sequelae similar to those observed for human survivors of NiV encephalitis [132]. In summary, the ability to efficiently evade the host’s innate immune response might be a major determinant of henipavirus pathogenicity. Like NiV and HeV, the M protein of CedV and GhV mediates the suppression of IFN expression via Iκκε [122], yet data on other novel henipa-like viruses are missing. As shown in Table S2, there is a lack of data regarding the ability of the novel henipa- and henipa-like viruses to block the interferon response. This highlights the need for more research on these viruses to assess their pathogenic potential for humans.

## 7. Tools to Study Newly Discovered Henipaviruses

Research on newly emerging henipaviruses is hampered by the lack of viral isolates and the potential need to perform infection studies at the highest biological safety level, BSL-4. Both NiV and HeV are classified as BSL-4 pathogens. They are also classified as Select Agents by the United States Federal Select Agent Program that is jointly comprised of the Centers for Disease Control and Prevention (CDC)/Division of Select Agents and the Toxins and the Animal and Plant Health Inspection Service/Division of Agricultural Select Agents and Toxins. In contrast to NiV and HeV, CedV is highly attenuated in various animal models. The Biosafety in Microbiological and Biomedical Laboratories manual published by the CDC and National Institutes of Health recommends performing work with CedV in tested, asymptomatic animal models at BSL-2 and work with new animal models at BSL-3, until it is demonstrated that CedV does not cause disease in these models [133]. Since no pathogenicity data are available for the newly discovered henipaviruses, work with infectious viruses should consequently be performed at BSL-4, limiting urgently needed research activity. To mitigate these research constraints, surrogate systems must be established to study the various steps of the viral replication cycle and test antivirals and therapeutic antibodies. The systems described below have been established for NiV, and to a lesser extent HeV, but research tools used to study the newly discovered henipa- and henipa-like viruses are still limited.

### 7.1. Tools to Study Henipavirus Replication and Transcription: Minigenome Systems

**Figure 4:**
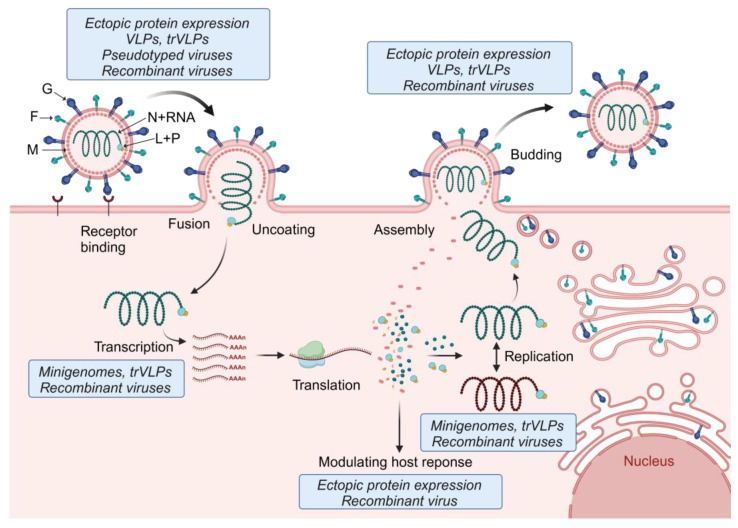
Tools used to study the different steps of the henipavirus replication cycle. The first step of the henipavirus replication cycle is G-mediated attachment and binding to the cellular receptor (e.g., ephrin-B2 or -B3). Fusion of the viral membrane with the cell membrane occurs at the cell surface and is mediated by a concerted effort of both G and F. The helical nucleocapsid complex is then released into the cytoplasm of the infected cell where primary transcription is initiated. The viral mRNAs are translated, leading to secondary transcription and viral genome replication. Replication takes place in cytoplasmic viral inclusion bodies. Some of the viral proteins, including P, V, W, and C, modulate antiviral host responses. Together with N, P, and L, the newly synthesized viral genomes are packaged into nucleocapsids and transported to the plasma membrane for viral particle assembly. Budding and viral particle release is mediated by M. The depiction of the henipavirus replication cycle was inspired by [146]. Tools used to study the various steps of the henipavirus replication cycle are indicated in the blue boxes. F, fusion glycoprotein; G, attachment glycoprotein; L, RNA-dependent RNA polymerase; M, matrix protein; N, nucleocapsid protein; P, phosphoprotein; trVLPs, transcription- and replication-competent virus-like particles; and VLPs, virus-like particles. Created with BioRender.com.

Transfection-based minigenome systems are useful tools to study both cis-acting signals in the viral RNA genome and protein factors required for genome replication and transcription under BSL-2 conditions. Minigenome systems have been established for many negative-sense RNA viruses, including henipaviruses. While most of the minigenome systems have been established for NiV, recent work also describes minigenome systems for HeV, CedV, and GhV [134,135]. Monocistronic henipavirus minigenomes typically consist of the 3′ and 5′ ends of the viral genome, which contain the encapsidation signals and the transcription and replication promoters. All viral genes are removed and replaced by a reporter gene that is flanked by virus-specific gene start and gene end signals. These signals determine initiation and termination of mRNA synthesis by the viral RNA-dependent RNA polymerase. The minigenome cDNA is cloned into a plasmid under the control of a DNA-dependent RNA polymerase promoter, such as T7 RNA polymerase (T7) or RNA polymerase I [60,134,135,136]. Precise minigenome ends can be generated with the help of ribozymes [60,135,137]. The minigenome plasmid is used to transfect cells, along with expression plasmids encoding the so-called support proteins, L, N, and P. If the minigenome (and the support proteins) are under the control of the T7 promoter, T7 must be co-expressed, either by co-transfecting a T7-encoding plasmid or by using a T7-expressing cell line. The successful replication and transcription of the minigenome by the support proteins can be measured by reporter gene activity. Frequently used reporter proteins include chloramphenicol acetyl transferase, luciferases, and fluorescent proteins [60,62,137,138,139,140,141]. A special feature of NiV minigenome systems is the use of mutant versions of the P gene to suppress the expression of C, V, and W [60,136]. Further developments include the generation of bicistronic minigenomes, the generation of monocistonic minigenomes that express two reporter genes from a single ORF, the use of codon-optimized support plasmids, and the generation of cell lines stably expressing NiV N, P, L, and the minigenome [141,142,143]. NiV minigenome systems were instrumental to reveal the structure of the bipartite replication promoter, to confirm that the rule of six also applies to NiV, to assess the function of noncoding regions, and to perform functional analyses of NiV N, P, and L gene products [60,62,138,140,142,143,144,145] (Figure 4). They have also been used to determine the polymerase function of heterologous support proteins [60,134,135] and for antiviral drug screening [134,136,141].

Developing minigenome systems for the newly discovered henipaviruses might be hampered by the lack of sequence information for the genome termini. Recent work on GhV showed that complementing the missing genome ends with the homologous sequences from HeV or NiV led to minigenome activity. However, minigenome activity was low compared to the NiV, HeV, and CedV minigenome systems that were established in parallel [134]. There are various possible explanations for this result. First, GhV replication and transcription could be generally slow compared to NiV, HeV, and CedV. Second, GhV replication could be inefficient in cells that are not derived from the natural reservoir host of this virus. Third, the complemented genome ends are incorrect and therefore viral genome replication and transcription are inefficient. Fourth, there might be sequencing errors in the published GhV genome sequence that interfere with efficient replication and transcription activity. This highlights one of the major challenges in working with newly discovered henipaviruses. Sequence information for each of these viruses is limited, and potential sequencing errors might interfere with establishing surrogate systems as well as generating recombinant viruses to study the various steps of the viral replication cycle and test antiviral countermeasures. Except for GhV, there are no minigenome systems available for any of the newly discovered henipa-and henipa-like viruses.

### 7.2. Tools to Study Entry, Fusion, and Viral Protein Function: Virus-like Particles, Pseudotyped Viruses, and Ectopic Protein Expression

While monocistronic minigenomes are instrumental to study viral replication and transcription mechanisms, other steps of the viral replication cycle, such as entry, fusion, and budding, cannot be analyzed. To overcome these limitations, a variant of the minigenome system, dubbed the transcription- and replication-competent virus-like particle (trVLP) system, was developed that allows studying all steps of the viral replication cycle (Figure 4). In addition to a reporter gene, the trVLP minigenome also encodes the genes required for viral entry, particle formation, and budding. This approach was first established for the Ebola virus [147]. trVLP systems for henipaviruses are based on a tetracistronic minigenome encoding M, F, and G in addition to a reporter gene. Upon transfection of cells with a plasmid encoding the tetracistronic minigenome along with the support plasmids (encoding N, P, and L), M, F, and G are expressed in the transfected cells, leading to the formation of virus-like particles (VLPs) that package the tetracistronic minigenome and can be used to infect cells. trVLP systems have been established for NiV, HeV, CedV, and GhV [134,148]. Since N, P, and L must be provided in trans to keep the trVLP systems up and running, they do not result in the production of infectious viruses and can be handled at BSL-2.

A simpler strategy to investigate viral entry, fusion, and budding is the use of VLPs and pseudotyped viruses expressing G and F (Figure 4). A recent review summarizes the use of these systems to determine henipavirus receptor usage, fusion determinants, the screening of neutralizing antibodies, and testing of antivirals [149,150]. VLPs were also instrumental in determining the role of the M protein in viral particle formation and budding. The expression of M with or without F and G led to the formation and release of VLPs that are morphologically similar to NiV particles [151].

Receptor binding and fusion can also be studied by overexpressing F and G in cells and determining the rate of cell-to-cell fusion (Figure 4). This strategy combined with protein purification and surface plasmon resonance was used to show that MojV and LayV G do not bind to the known henipavirus receptors ephrin-B1, -B2, and -B3 [94,98]. In addition, GhV F was shown to efficiently mediate fusion in cells derived from the fruit bats *Hypsignathus monstrosus* and *Eidolon helvum* but not in human cells [90,91,92,93]. This highlights the need for access to cells from the natural reservoir hosts of zoonotic viruses to be able to identify correlates of host tropism, including receptor usage and fusion activity. In addition to studying viral entry and particle release, ectopic protein expression has been used to analyze virus–host interactions, including the immune modulatory functions of henipaviral proteins (Figure 4; see also Section 6 and Table S2). Although NiV and HeV proteins inhibit the IFN response in both human and fruit bat cell lines [152,153], differences in ISG expression were observed between these cell types [154,155]. Having access to diverse cell types from the reservoir hosts of the various henipaviruses is essential for uncovering differences in the host immune response compared to human cells. Although immortalized cell lines derived from the host species are of great value, they might not recapitulate the innate immune responses observed in primary cells. Novel approaches based on reprogrammed stem cells from fruit bats [156] or bat–mouse bone marrow chimera models [156] will provide important research tools to identify correlates of protection in the natural reservoir hosts.

### 7.3. Virus Rescue Systems

Rescue systems used to generate recombinant, infectious henipaviruses work similarly to minigenome systems. Instead of a minigenome plasmid, a plasmid containing a full-length cDNA copy of the viral antigenome is transfected along with the plasmids encoding the support proteins (N, P, and L), leading to the production of infectious viruses. Virus rescue systems can be used to introduce mutations into the viral genome and to insert additional transcription units, including genes encoding reporter proteins. Recombinant virus rescue systems have been established for NiV [157], HeV [157], and CedV [129]. For CedV, generating recombinant viruses from cDNA made it possible to perform work at a lower biosafety level after it was shown that CedV is highly attenuated and does not cause disease in various animal models. The original CedV isolate was handled in a high containment laboratory and had to remain there [129]. Because of its close relationship to HeV and NiV, recombinant CedV has been used as a surrogate virus to identify antivirals against pathogenic henipaviruses under BSL-2 conditions [158]. To mimic NiV and HeV entry, recombinant chimeric Cedar viruses were generated expressing the NiV or HeV F and G proteins. The chimeric viruses bound to the NiV and HeV entry receptors and were utilized to test neutralizing antibodies and entry inhibitors at a lower biosafety level [159]. Rescue systems can also be used to generate recombinant viruses based on sequence information if there are no viral isolates available [160]. While rescue systems have not been established yet for any of the newly discovered henipa- and henipa-like viruses, they would be useful tools for pathogenicity studies and the development of antiviral countermeasures (Figure 4). As mentioned above, a potential challenge of generating recombinant henipa- or henipa-like viruses is the lack of sequence information, specifically missing genome end sequences.

## 8. Conclusions: Do the Novel Henipaviruses Pose a Risk to Human Health?

While MojV was discovered in rats in an abandoned mine in China following an outbreak of fatal pneumonia with an unknown cause among miners who had worked in the mine, it is not clear if MojV was the etiological agent causing the disease [45]. LayV is currently the only newly identified henipa-like virus with verified zoonotic spillover into humans. There were no reported fatalities among the 35 identified patients with acute LayV infection, and there was no evidence for human-to-human transmission [48]. The potential mechanism of zoonotic spillover of LayV to humans remains unclear.

Although serological evidence suggests there were past spillover events of henipaviruses into humans in Cameroon, which may contribute to the prevalence of undiagnosed or misdiagnosed encephalitis cases, it is not known if GhV causes human disease [161]. In the absence of virus isolates, studies using different cell lines transiently expressing recombinant GhV F and G reported that syncytia formation was limited to bat-derived cell lines [90,91,92], as mentioned in Section 5. However, these experiments are not as informative as data acquired with an isolated infectious virus. Serologic screenings of domestic animals in Ghana suggest that pigs have been exposed to henipa-like viruses close enough to NiV and HeV to generate cross-reacting but not cross-neutralizing antibodies, highlighting the risk of zoonotic spillover in Africa. Notably, it is not known if these spillover events were caused by GhV or by another African henipa-like virus, as RNA of at least 19 different species of henipa-like viruses has been found in bat samples from Western and Southern Africa, with GhV being the only representative of which the full genome was sequenced [44].

Data on how the novel henipa- and henipa-like viruses modulate the human innate immune system are still sparse. Although no human infections with henipa-like viruses have been reported in Republic of Korea, isolated GAKV was shown to infect human lung epithelial cells, again highlighting the risk of potential spillover events [50]. Little is known about the abilities of the newly discovered henipa-like viruses to interfere with the human immune response, and more research is needed to shed light on this interaction and the concomitant risk these viruses may pose to human health.

It should be noted that this review primarily focuses on newly discovered henipa- and henipa-like viruses that were published in the literature. However, numerous additional henipa-like viral genome fragments have been deposited to GenBank in recent years. This highlights the high prevalence and widespread geographic distribution of henipa- and henipa-like viruses, and it is to be expected that the discovery of further novel viruses belonging to this group will continue in the near future. While many of these viruses differ from NiV and HeV in various aspects, including animal reservoir, geographic distribution and, importantly, entry receptor usage, the case of LayV highlights that viruses from the newly discovered shrew- and rodent-borne clade can infect and cause disease in humans. Therefore, the emergence and pathogenic potential of novel henipa-like viruses must be closely monitored to enhance our preparedness for potential future outbreaks.

## Supplementary Materials

The following supporting information can be downloaded at: https://www.mdpi.com/article/10.3390/pathogens13070587/s1, Table S1: Overview of the currently classified henipaviruses and unclassified henipa-like viruses; Table S2: Interaction between henipavirus proteins and immune signaling pathways.
