# Supplementary material for: A Comparative Assessment of the Pathogenic Potential of Newly Discovered Henipaviruses
Source: Pathogens. 2024 Jul 16;13(7):587. doi: 10.3390/pathogens13070587 (PMC11280395; doi:10.3390/pathogens13070587)
Supplement: Supplementary file 1 [file pathogens-13-00587-s001.zip › pathogens-3036733-supplementary.pdf]

pathogens

MDPI

Review

Comparative assessment of the pathogenic potential of newly discovered henipaviruses

Kristina Meier, Judith Olejnik, Adam J. Hume, and Elke Mühlberger

Supplementary data

Table S1: Overview of the currently classified henipaviruses and unclassified henipa-like viruses. Classified henipaviruses are highlighted in yellow, unclassified henipa-like viruses in blue. Please note that there will always be a time lag between virus discovery and classification. Thus, the classification status of viruses listed here as unclassified may change soon. Virus abbreviations provided in Figure 1.

	Virus	Host species	Year of discovery	Known distribution	Pathogenic potential
Henipaviruses	HeV	*Pteropus* fruit bats	1994	Northeastern Australia	Severe in humans
	NiV	*Pteropus* fruit bats	1999	South/Southeast Asia	Severe in humans
	CedV	*Pteropus* fruit bats	2009	Northeastern Australia	Nonpathogenic in humans
	GhV	*Eidolon* fruit bats	2009	Ghana	Unknown
	MojV	*Rattus* rodents	2012	Southwestern China	Not verified
Unclassified henipa-like viruses*	AngV	*Eidolon* fruit bats	2019	Madagascar	Unknown
	LayV	*Crocidura* shrews?	2018	Eastern China	Moderate in humans
	GAKV	*Crocidura* shrews	2017	South Korea	Unknown
	DARV	*Crocidura* shrews	2017	South Korea	Unknown
	MeliV	*Crocidura* shrews	2018	Guinea	Unknown
	DewV	*Crocidura* shrews	2019	Belgium	Unknown
	NinExV	*Sorex* shrews	2020	Belgium	Unknown
	PBV	*Marmosa* opossum	2015	Brazil	Unknown
	SCtV	*Crocidura* shrews	2021	Central China	Unknown
	ResV	*Crocidura* shrews	2019	Germany	Unknown
	HasV	*Crocidura* shrews	2020	Germany	Unknown
	JCsV	*Crocidura* shrews	2016	Central China	Unknown
	JCs2V	*Crocidura* shrews	2016	Central China	Unknown
	LechV	*Crocidura* shrews	2021	Germany	Unknown
	WSmV	*Suncus* shrews	2016	Eastern China	Unknown
	WAaV	*Apodemus* rodents	2016	Eastern China	Unknown
	WCaV	*Crocidura* shrews	2016	Taiwan	Unknown
	WCsV	*Chodsigoa* shrews	2016	Taiwan	Unknown
	SChV	*Chodsigoa* shrews	2022	Southwestern China	Unknown

Table S2: Interaction between henipavirus proteins and immune signaling pathways.

Cellular target	NiV	HeV	CedV	GhV	Other henipa- and henipa-like viruses
RIGI-like receptors	V	V	V not expressed	ND	ND
Iκκα/β dimerization/IRF activation	C	ND	ND	ND	ND
TRIM6 /Iκκε activation	M	M	M	M	ND
IRF3 activation/IFN expression	V	ND	V not expressed	ND	ND
	W	W	W not expressed		
STAT 1/2 activation/nuclear translocation	P	ND	P not inhibiting	ND	ND
	V	V	V not expressed		
	W	W	W not expressed		
	N	N	ND		
	M	ND	ND		

ND = no data available.
